# Postprandial Hypotension: An Underreported Silent Killer in the Aged

**DOI:** 10.7759/cureus.35411

**Published:** 2023-02-24

**Authors:** Ayoola Awosika, Uzochukwu Adabanya, Richard M Millis, Adekunle E Omole, Jin Hyung Moon

**Affiliations:** 1 College of Medicine, University of Illinois, Chicago, USA; 2 College of Health Sciences and Professions, Ohio University, Athens, USA; 3 Community Medicine, Mercer University School of Medicine, Columbus, USA; 4 Pathophysiology, American University of Antigua, St. John's, ATG; 5 Anatomical Sciences, American University of Antigua, St. John's, ATG; 6 General Medicine, Mercer University School of Medicine, Columbus, USA

**Keywords:** digestion, blood glucose, hypotension, blood pressure, syncope, autonomic failure, geriatrics

## Abstract

Orthostatic hypotension (OH) is one of the most common autonomic dysfunctions, with high prevalence in populations of elderly, hypertensive, diabetic, or Parkinson’s patients. Evidence is emerging that OH co-occurs with postprandial hypotension (PPH); a greater prevalence of PPH than of OH is reported for Parkinson’s disease patients. OH is diagnosed by measuring the blood pressure changes associated with postural changes and often produces alterations in consciousness or other such bothersome symptoms as fainting. PPH is diagnosed by measuring the blood pressure changes associated with ingesting high carbohydrate test meals. Because of the time lag between food ingestion and absorption, PPH is often not reported as symptomatic and, therefore, not diagnosed as PPH. OH and PPH are independent predictors for all causes of mortality. Relative underdiagnosis may qualify PPH as a “silent killer” disease. This review is aimed at providing updates on the epidemiology, pathophysiology, and clinical aspects associated with the diagnosis and treatment of PPH. Highlighting the current gaps in knowledge and research about PPH is expected to make medical practitioners more cognizant of the dangers of underdiagnosis and motivate future research to identify individuals and populations at high risk for PPH and its sequelae.

## Introduction and background

History, definition, and epidemiology of postprandial hypotension

In 1935, Gladstone identified and described a rare condition he observed in a hypertensive patient whose blood pressure had decreased from 185/120 mmHg to 140/80 mmHg after a meal [1]. Later in 1953, Smirk also reported a decrease in BP after eating in patients with autonomic failure [2]. Further to this, Robertson and his colleagues noticed that patients who suffered from chronic autonomic failure exhibited a significant fall in systolic blood pressure (BP); specifically, a 49±6 mmHg decrement after eating [1]. As the years evolved, postprandial hypotension (PPH) was officially recognized as a medical problem in 1977 [1].

In recent years, the diagnosis of PPH is increasing among nursing home residents [3] likely because of an aging geriatric population requiring round-the-clock care. Many scholars define PPH as a fall or decrease of at least 20 mmHg in the systolic BP within two hours after a meal [3]. The definition is comparable to that of postural hypotension. However, there is a limited understanding of PPH likely because it lacks a standardized definition. When the systolic BP decreases below 90 mmHg after eating, patients often have symptoms attributed to disrupted cerebral blood flow autoregulation [4]. The cumulative impact of multiple factors (i.e., medication administration, postural change, etc.) before, during, and after eating may influence the clinical assessment of a patient’s postprandial BP responses.

PPH is prevalent in approximately 24-33% of elderly populations receiving care in nursing homes, about 67% of geriatric patients, and approximately 50% of individuals suffering from unexplainable syncope [5]. Markedly, the disease is 33% prevalent among the healthy population [3]. Hypotension in response to oral glucose or ingesting mixed meals is prevalent among young and elderly, normotensive and hypertensive patients in nursing homes [3], and is highly prevalent in patients suffering from comorbid conditions such as cardiovascular disease, autonomic insufficiency, diabetes mellitus, paraplegia, and renal failure. Since PPH remains underdiagnosed, this review is aimed at highlighting the current gaps in knowledge and research about PPH while providing updates on the epidemiology, pathophysiology, and clinical aspects associated with the diagnosis and treatment of PPH.

## Review

Pathophysiology of PPH: causes and associations

The pathophysiology of PPH is multifactorial, mainly involving sympathetic dysfunction associated with autonomic neuropathy [6]. Patients with Parkinson's disease, diabetes mellitus, and heart failure often lack the capacity to increase their cardiac output or have an attenuated baroreceptor reflex [6]. Persons with an attenuated baroreflex are incapable of increasing their heart rate in response to an acute fall in BP when there is increased splanchnic blood flow resulting from vasodilation to the gastrointestinal tract organs after a meal. Food ingestion is known to cause the pooling of splanchnic blood because of the larger amounts of blood required for gastrointestinal motility, digestion, and absorption. This splanchnic vasodilation reduces venous return, stroke volume, and cardiac output, and this activates the baroreflex mechanism to increase systemic (total peripheral) vascular resistance and heart rate to maintain normal BP [4]. These responses do not occur among older adults suffering from impaired sympathetic reflex activity or patients living with autonomic nervous dysfunction, thereby leading to PPH [7].

Insulin is implicated in the pathogenesis of PPH by the mechanism of vasodilation and hypotension reported in diabetic patients after an acute administration of insulin [8]. The genetic susceptibility to postprandial blood pressure dysregulation remains poorly understood. Though some studies have shown an association between polymorphisms of beta-adrenergic receptor (β-AR) genes and orthostatic blood pressure dysregulation in hypertensive patients [6,7], more research is needed to characterize any genetic predisposition associated with PPH. A summary of the pathophysiologic mechanisms is found in Table 1.

**Table 1:** Potential causative mechanisms

Potential mechanisms	1. Increased splanchnic blood pooling
	2. Attenuated baroreflex function due to age- or hypertension-related impairment
	3. Inadequate sympathetic nerve firing or vascular responsiveness to norepinephrine
	4. Upregulation of vasoactive intestinal peptides
	5. Insulin-mediated vasodilation

PPH state associated with youth, aging, and autonomic failure 

Studies have shown that young healthy subjects have modest cardiovascular vasodepressor responses to eating [9]. Ingestion of food increases bowel volume and diverts blood to the mesenteric vessels. In turn, there is a baroreceptor-mediated sympathetic reflex response, which includes increased heart rate, cardiac output, and total peripheral resistance. These effects are accompanied by vasoconstriction of several peripheral vascular beds including skeletal muscle. Plasma norepinephrine (NE) is reported to remain constant due to effective reuptake and clearance of excess NE from the nerve terminal; hence, there is no spillover of NE into the plasma [9]. Overall, this culminates in a slightly increased or stable systolic BP.

In elderly healthy subjects, hemodynamic responses to eating are generally more pronounced compared to young healthy subjects. There appears to be an age-related increase in the release of NE, which causes spillover from the sympathetic nerve terminals into the blood plasma. The heart rate response is shown to be blunted due to an age-related decline in beta-adrenergic responsiveness [10]. Thus, meal stimuli appear to create greater physiologic stress in the elderly despite a stable BP.

Table 2 summarizes the hemodynamic mechanisms involved in physiological and pathophysiological postprandial responses, including PPH associated with autonomic failure.

**Table 2:** Age-dependent physiologic and pathophysiologic postprandial responses ↑ : increase ↔ : Normal ↓ : Decrease Information from: Lipsitz et al., 1993 [9]

	Young Healthy	Elderly Healthy	Autonomic Failure (PPH)
Heart rate	↑	↑	↑ ↑
Blood pressure	↔	↔ or ↓	↓ ↓
Plasma norepinephrine	↔	↑	↔
Vascular norepinephrine responsiveness	↑	↔ or ↓	↓↓

In contrast to young and elderly healthy subjects, elderly patients with autonomic insufficiency have difficulty maintaining postprandial BP, which is thought to result from inadequate sympathetic nerve activity and/or synaptic release of NE. Elderly patients with previously mentioned conditions associated with varying degrees of autonomic failure (e.g., Parkinson’s disease, diabetes, and heart failure patients) exhibit inappropriate vascular responses to NE release after eating. This is evidenced by impaired peripheral vasoconstriction leading to reduced systemic vascular resistance, cardiac index, and a profound decrease in BP [11]. It is noteworthy that an unexplained, paradoxical cardio-acceleration of heart rate, which fails to increase BP, is reported in healthy elderly subjects and elderly patients with the previously mentioned conditions of autonomic failure postprandially. We speculate that this cardio-acceleration may result from a combination of parasympathetic withdrawal and/or sympathetic overdrive, possibly mediated by gastrointestinal neurohormonal mechanisms involving histamine, vasoactive intestinal peptide, etc.).

Symptoms and clinical presentation

Much about PPH is speculative because there is a paucity of research, and the scientific literature describing PPH suffers from small sizes of cohorts, the absence of appropriate control subjects, the lack of longitudinal assessments, and the presence of numerous potential cofounders. Hence, a more in-depth, larger-scale approach to research will probably be needed to derive a valid instrument for diagnosing and assessing patients suspected of having PPH.

Although many patients may be asymptomatic, the most common signs and symptoms of PPH include motor weakness, dizziness, light-headedness, syncope, falls, angina pectoris, nausea, and visual disturbances such as black spots in the visual field (“floaters”). Patients may also be unable to walk or stand after meal consumption [3]. Case reports of PPH have described transient ischemic attacks in older patients presenting with large postprandial decrements in BP, with symptoms often resolving when the BP returns to normal. Cerebral symptoms are subject to or dependent on the characteristics of the cerebral hypoperfusion (e.g., white vs. grey matter, cortical or brainstem sites [5]. It is noteworthy that PPH is reported less frequently in elderly patients that have experienced a fall compared to those that have not experienced a fall [12]. This may be due to other potential confounding co-morbidities that seem to exist in the elderly population.

Diagnosis of PPH

Clinicians should suspect PPH specifically in the elderly with diabetes, Parkinson's disease, end-stage renal disease, and heart failure patients [13]. Inquiring about these patients' hypotensive symptoms after meal ingestion is of paramount importance in correctly deriving the diagnosis of PPH. All symptomatic patients should be subjected to BP monitoring, and clinicians should be prompted to carefully document the systolic BP after every meal.

The diagnosis of PPH is preferably done by using home monitoring BP devices. While an argument could be made for the use of ambulatory BP monitoring, the cost and patient tolerance for home monitoring BP devices are far more advantageous. For the purposes of diagnosis, the test can be done in a nursing home facility or clinic setting by placing the patient in a supine position. Check the baseline BP and heart rate after five minutes of rest before administering the test meal or food or beverage (pre-prandial) [14]. Once the mixed liquid meal is administered, measure the patient’s BP and heart rate every 10 min for about two hours using an automated sphygmomanometer for reproducibility [15]. When an assessment is done with an ambulatory monitor, measurements should be performed every 15 minutes throughout the day and every 50 minutes at night [15]. The required, diagnostic BP drop (≥20mm Hg drop in systolic blood pressure) can usually be recognized as soon as 15 minutes after eating in 15% of PPH patients; can be detected in about 70% of patients at 30-60 minutes, postprandially [14]. A few patients, however, are reported to experience the required, decrement in BP at 75-120 minutes after eating [15]. No specific meal or caloric restrictions have been reported so far for the test procedure, but it may be preferable to use test meals that are low in carbohydrate content due to the influence of insulin-induced reactive hypoglycemia, which may necessitate concomitant blood glucose monitoring.

**Figure 1:**
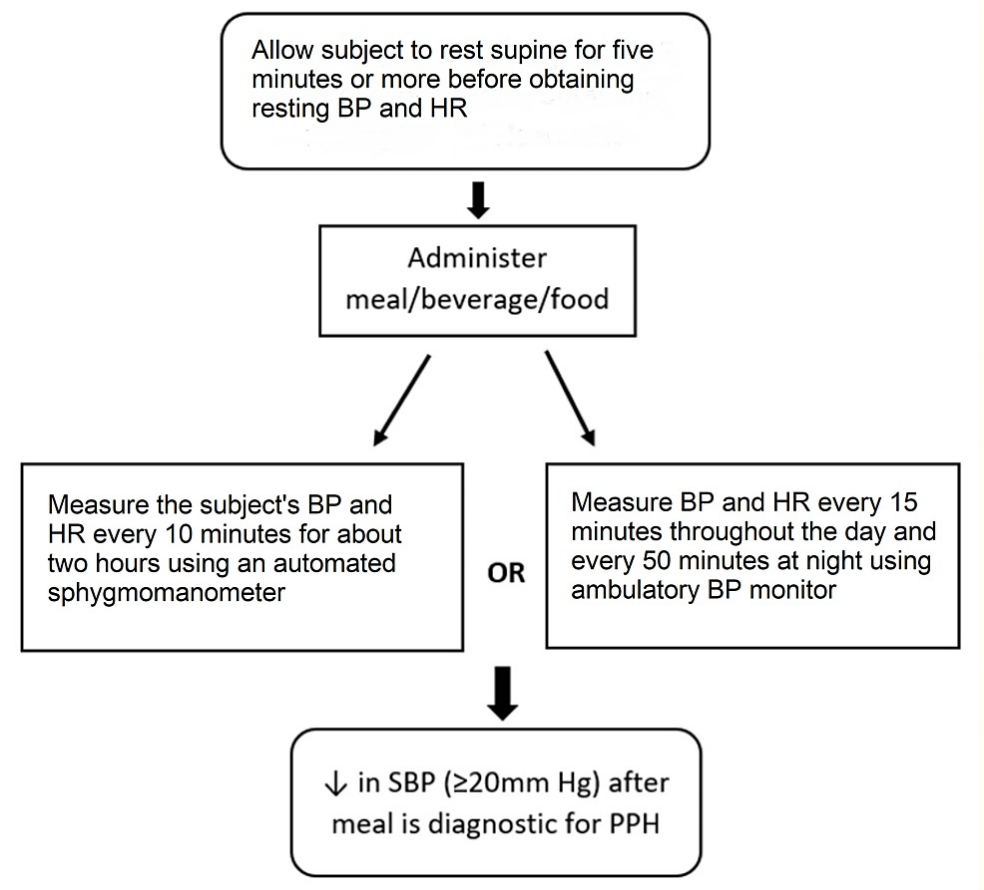
Postprandial hypotension diagnostic steps PPH: postprandial hypotension; BP: blood pressure; HR: heart rate; SBP: systolic blood pressure

The intraindividual reproducibility of PPH appears to be relatively high; therefore, a single test is often sufficient for correctly diagnosing the condition [14]. The diurnal variability of PPH in the elderly is not known; however, postprandial BP responses appear to be greater in the mornings and lessened, with fewer symptoms reported, in the later hours of the day than in the mornings [14]. These findings suggest that diagnostic procedures performed in the mornings may be more productive and adjusting the daily living activities and administration of BP-lowering medications to the later hours of the day when episodes of PPH are known to be least prevalent should mitigate the risk of developing symptomatic PPH in affected patients. A summary of the diagnostic steps can be found in Figure 1.

Medical management strategies for PPH

The complex epidemiology and pathogenesis of PPH create difficulties in achieving satisfactory results with pharmacological treatment alone. Hence, clinicians need to be cognizant of both the pharmacological and the nonpharmacological options for the treatment of patients diagnosed with PPH.

Nonpharmacologic Interventions

One of the primary treatment strategies is to reduce gastric activity to delay exposure of the products of digestion to the small intestine. Delayed emptying with moderate gastric distension is shown to result in a 200% increase in sympathetic nerve activity [1,4]. Such an increase in sympathetic signaling may be effective in maintaining postprandial BP. Hence, eating smaller meals more frequently and drinking more water also contributes to protective gastric distension and slowing stomach emptying.

Drinking water before meals may help reduce the drop in postprandial BP that characterizes the condition. Drinking 350-480 mL of water is reported to result in a 20 mmHg gain in BP in patients experiencing autonomic failure [16,17]. Improvement in PPH in patients with autonomic failure is reported when six small meals were consumed compared to three large meals. The smaller meals were associated with less than 11-20 mmHg reduction in postprandial BP [18].

Ten minutes of postprandial walking (mild intensity, aerobic exercise), commencing about 20 mins after breakfast was shown to be temporarily effective in about 14 feeble elderly patients. Mean arterial pressure increased by 18±4 mmHg during post-prandial exercise but dropped to pre-exercise levels 10 mins after exercise ceased [18,19]. This suggests that postprandial exercise may be of benefit in the prevention and management of PPH.

Studies have shown that cold glucose loading caused an increase in mean arterial pressure by a maximum of 3.9±1.3 mmHg (p<0.01), while warm glucose loading decreased BP by a maximum of 8.0±1.1 mmHg (p<0.001); indicating that the modification of the meal temperature may be adopted as a form of non-pharmacologic interventions to control PPH [20].

Pharmacologic Interventions

Studies related to treating the condition with pharmaceuticals involve a variety of approaches. Somatostatin analogs, vasopressin, non-steroidal anti-inflammatory drugs (NSAIDs), and other agents like caffeine have been used.

Caffeine, an adenosine receptor antagonist has been used for stimulation of the sympathetic nervous system. Caffeine maintains BP by constricting arterial blood vessels [21]. Caffeine should be administered before day-time meals (breakfast or lunch) and avoided at night-time (dinner) to prevent sleep disruption seen in many patients. Although there have been conflicting study results about caffeine use, titrating between 60 mg and 200 mg before a meal appears to prevent PPH in some patients. A 4 mmHg gain in BP of elderly patients who ingested 60 mg of caffeine (as tea or coffee) five times daily without affecting their baseline systolic BP [22]; 200 mg of caffeine is reported to prevent PPH in elderly subjects who previously exhibited 14 mmHg reductions in postprandial BP [23].

The administration of somatostatin analogs such as octreotide may be used for local vasoconstriction to reduce splanchnic blood flow, thereby redistributing blood flow from the splanchnic to the central circulation for maintaining postprandial BP [24]. Octreotide has been used to treat carcinoid tumors, vasoactive intestinal peptide-secreting adenomas, acromegaly, and diarrhea [25].

Acarbose is an alpha-glucosidase inhibitor that may be useful for delaying gastric emptying. Acarbose functions by inhibiting the enzymes required for carbohydrate digestion, thereby decreasing the number of particles created as products of digestion transported into the duodenum after a meal [26]. Acarbose has the potential to be effective because the dumping of carbohydrate products into the duodenum is a major factor in speeding up gastric emptying, whereas the products of lipid and protein digestion tend to slow gastric emptying [26]. Inhibiting the enzymes of carbohydrate digestion in the stomach is thought to reduce the release of gastrointestinal peptides such as vasoactive intestinal peptide (VIP) that are known to mediate splanchnic vasodilation [27]. Meta-analysis reports show that acarbose attenuates the fall of postprandial systolic and diastolic BP and is, therefore, effective in preventing PPH [26].

The mechanism of the blood pressure increase by NSAIDs is not fully understood, and NSAIDs should typically be avoided in the elderly due to an increased risk of accidental falls. NSAIDs have also been shown to be associated with gastric ulcers and many other side effects common to the elderly. Many elderly patients also have comorbid conditions and are therefore on medications that may exhibit drug interactions with NSAIDs. Prophylactic use of NSAIDs for the prevention and control of PPH is therefore not advisable for most geriatric patients. NSAID use for PPH is not fully understood; however, by implication, the outcome is most likely due to its effect on prostaglandin metabolism. Prostaglandin increases renal blood flow and the glomerular filtration rate (GFR). NSAIDs, which inhibit the production of prostaglandin, can prevent PPH likely by such renal mechanisms that decrease renal blood flow, increase sodium retention, circulating blood volume, cardiac preload, and, therefore, systemic BP [28].

Calcitonin gene-related peptide (CGRP) is shown to play a role in PPH by virtue of its strong vasodilatory activity in response to glucose loading [29], thereby suggesting a role for the new class of anti-migraine CGRP inhibitor drugs in PPH. Indeed, headaches are common sequelae of low BP associated with OH and other causes as well [30]. 

Psychosocial and lifestyle considerations for PPH

In patients with PPH, large meals are more likely to result in a significant drop in BP and peripheral vascular resistance than small meals [30]. It is, therefore, advisable to regulate the food intake of PPH patients using small portions with more frequent eating than two to three large meals per day, as is common in most nursing homes and assisted living residences for the elderly. Symptomatic patients should also rest by lying supine after meals because standing or sitting tends to have an additive hypotensive effect [31]. Ambulating appears to aid in restoring BP after meals, especially in nursing home settings [4]. Dehydration can also predispose patients to PPH and can be prevented through adequate intake of fluids among susceptible persons. As an individual advances in age, water, and renal salt conservation decrease, making the elderly vulnerable to hypovolemia. Some elderly community members also restrict their salt consumption, reducing the circulating blood volume, and thereby creating conditions for PPH [28]. Hot weather can also make the at-risk populations more vulnerable to PPH by reducing circulating blood volume, a result of heat-induced cutaneous vasodilation and sweating.

Complications of PPH

Due to inefficient cardiovascular response, there is an increased risk for cerebrovascular disease, transient ischemic attack, syncope, falls, and coronary events. A prospective study conducted on geriatric hypertensives using magnetic resonance imaging of the brain reported that 83% of hospitalized patients with episodes of PPH have evidence of lacunae that are suggestive of cerebrovascular damage [32]. Coronary events like angina could also be exacerbated due to increased cardiac workload from the autonomic response. The coronary blood vessels are also sensitive to freely circulating vasoconstrictor substances that are released in response to food ingestion [13].

Other considerations for clinical practice and patient education

**Figure 2:**
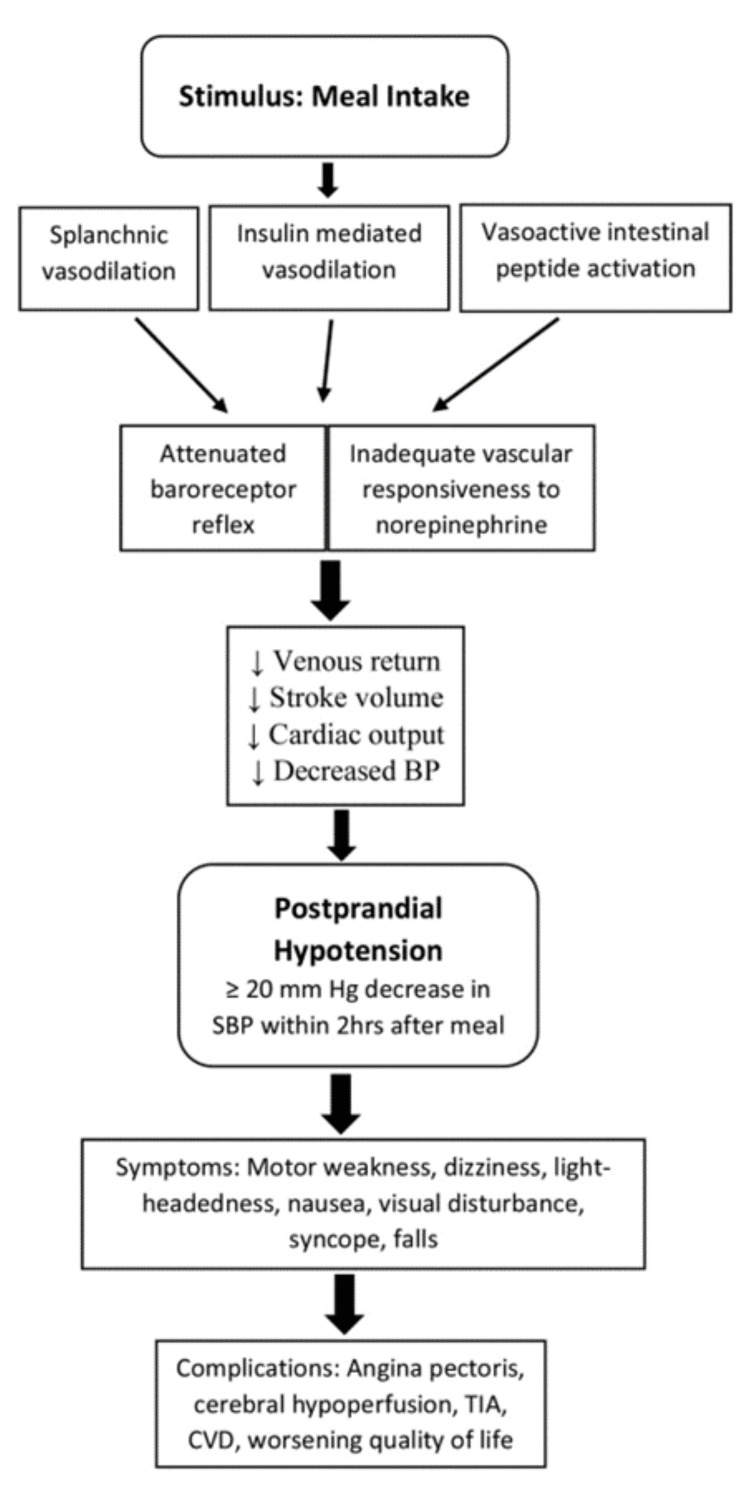
Summary of PPH mechanisms, symptoms, and potential complications ↓: decrease PPH: postprandial hypotension; BP: blood pressure; SBP: systolic blood pressure; TIA: transient ischaemic attack; CVD: cardiovascular disease

There are patients who may have other co-morbidities to watch out for when screening, diagnosing, and treating for PPH. These conditions can worsen the patient’s quality of life and response to treatment. Cases of patients with diabetic neuropathy may exhibit poor baroreceptor sensitivity and, therefore, autonomic unresponsiveness. This may potentially result in marked PPH. On the other hand, the administration of exogenous insulin can also exacerbate PPH. Hence, there is a need to titrate insulin to tolerable doses based on a personalized therapeutic range. And at the same time, it’s essential to counsel patients appropriately on anticipatory complications. Other drugs like ganglionic blockers, anti-parkinsonism agents (levodopa), reserpine, and clonidine may also worsen PPH. Conversely, patients with dopamine beta-hydroxylase deficiency, a metabolic disorder, do not have the ability to convert dopamine into norepinephrine and epinephrine; hence, they can present with OH, but they are able to maintain their BP after meals. The same can also be said for tetraplegic patients with complete cervical cord transection [33,34]. A summary given in Figure 2 may be helpful to clinicians and patients in recognizing and developing a proactive preventive and therapeutic approach to PPH.

## Conclusions

PPH is loosely defined as a fall in systolic BP of at least 20 mmHg within two hours after a meal. A standardized definition of PPH is yet to be established. The precise pathophysiology of PPH remains undefined and is, therefore, based largely on extrapolation of knowledge about autonomic failure and OH. PPH appears to involve sympathetic dysregulation, primarily found in patients with diabetes, Parkinson’s disease, and multiple system atrophy. PPH can also develop in patients with an attenuated ability to increase their cardiac output because of heart failure or an attenuated baroreflex. PPH is posited to be influenced by eating large amounts of food in young people, lack of salt intake in the elderly, dehydration, and hot weather. Some people may also be predisposed to PPH genetically. Common PPH symptoms include dizziness, nausea, angina pectoris, weakness, falls, black spots in the visual field, syncope, and light-headedness. During the diagnosis, healthcare providers check the BP before and after meal ingestion at intervals of about 15 minutes. The illness can be treated pharmacologically, non-pharmacologically, or both, and involves the use of drugs such as NSAIDs, somatostatin analogs, and acarbose.
